# Chronic Lateral Ankle Instability in Highly Active Patients: A Treatment Algorithm Based on the Arthroscopic Assessment of the Calcaneofibular Ligament

**DOI:** 10.7759/cureus.14310

**Published:** 2021-04-05

**Authors:** Ioannis K Triantafyllopoulos, Dimitrios G Economopoulos, Andreas Panagopoulos, Louw van Niekerk

**Affiliations:** 1 5th Orthopaedics Department, Diagnostic and Therapeutic Centre of Athens - Hygeia, Athens, GRC; 2 Orthopaedics Department, Volos General Hospital, Volos, GRC; 3 Orthopaedics Department, Medical School, University of Patras, Patras, GRC; 4 Orthopaedics and Trauma Department, Ministry of Defence Hospital Unit (MDHU) Northallerton, Northallerton, GBR

**Keywords:** calcaneofibular ligament, chronic ankle instability, arthroscopy, athlete, brostrom-gould

## Abstract

Background

Ankle sprains are common injuries that may recur as chronic conditions. We aim to describe a treatment algorithm for chronic lateral ankle instability based on the arthroscopic findings of the calcaneofibular ligament (CFL).

Methods

We assessed 67 highly active patients with chronic lateral ankle instability. They were recreational athletes or active military personnel. After clinical examination, they were all investigated further with MRI scans and stress views. Diagnostic arthroscopy followed, where the integrity of the CFL was assessed. Patients with an intact CFL were placed in group A while those with CFL tears in group B. Concomitant intra-articular pathologies, if present, were treated arthroscopically. CFL tears mandated that modified Broström-Gould reconstruction would follow. The American Orthopaedic Foot and Ankle Society (AOFAS) and Tegner scores were noted post-injury and during the 24-month follow-up.

Results

A total of 37 patients were put in group A and 30 in group B. The posterior talofibular ligament was intact in both groups. Synovitis and scar tissue were more common in group A (p = 0.01) compared to group B. Overall, no postoperative ankle instability or relapsing ankle sprain was documented. Both groups demonstrated significant improvement in their Tegner (p = 0.009) and AOFAS scores (p = 0.001) during their 24 months follow-up. Inter-rater reliability for CFL tears was moderate on clinical examination (k = 0.514) and fair on MRI, in conjunction with ankle arthroscopy (k = 0.357).

Conclusion

Our proposed algorithm offered a reliable pathway for accurate evaluation and successful treatment of chronic lateral ankle instability in high-demand groups.

## Introduction

Lateral ankle sprains are the most common lower extremity injuries seen by healthcare providers [1]. More than half of these injuries occur after excessive ankle plantar-flexion and inversion during athletic activities or military training.

Even though ankle sprains are mostly inconsequential, some may recur as chronic occurrences. The literature suggests that the incidence of recurrent inversion sprains and chronic ankle instability varies between 20% and 40% [2]. Several methods of conservative treatment, such as peroneal muscle strengthening, ankle bracing that blocks subtalar joint inversion, and application of small lateral heel wedges, have been described. [3-5]. These methods offer good results even in very active groups such as athletes and military personnel.

Knowledge of regional anatomy is essential for investigating the severity of ankle sprains. Both the anterior talofibular ligament (ATFL) and calcaneofibular ligament (CFL) are considered the primary static restraints during ankle inversion [6]. Their integrity is assessed with anterior drawer and lateral talar tilt tests, respectively. Magnetic resonance imaging (MRI) also offers valuable information for the assessment of chronic ankle instability patients [7].

The ATFL is the primary stabilizer against excessive ankle inversion. It is the weakest ligament of the lateral ligament complex (LLC) and the first to be injured in ankle sprains [8]. The stress upon it increases as the ankle moves into plantar-flexion and inversion, whereas the addition of internal rotation amplifies its risk for tear.

The CFL is the only ligament bridging the tibiotalar and subtalar joints. It provides ankle and subtalar joint stability and acts as a pulley for the peroneal tendons during their course through the peroneal groove of the lateral malleolus. Its integrity is compromised when excessive stress is applied while the ankle is inverted and dorsiflexed.

The posterior talofibular ligament (PTFL) is of less clinical significance. Due to its size and thickness, it is rarely injured with excessive ankle inversion. However, even in cases where PTFL is ruptured, reconstruction is not necessary [9].

The pattern of lateral ankle ligamentous injury is well understood. There is an agreement that the anterior structures are the first to be disrupted and that the damage extends posteriorly as severity increases. Hence, the ATLF is the first ligament to be injured followed by the CFL and PTFL [10]. Moreover, this injury pattern precludes isolated CFL ruptures [11].

According to pathomechanics, chronic lateral ankle instability can be either "functional" or "mechanical". In "functional" ankle instability, patients report a subjective feeling of "giving way" with provocative tests being negative. Conversely, in "mechanical" instability, provocative tests are positive.

Conservative management is the mainstay of treatment in chronic lateral ankle instability. However, surgery may be an option in patients with mechanical instability who do not improve with nonoperative treatment. In such cases, the objective of operative treatment is to restore stability and decrease the risk of posttraumatic osteoarthritis.

Numerous operative techniques have been described for this type of injury. These are either anatomic or non-anatomic. The rationale of anatomic reconstruction is to restore anatomy with direct repair of the identified ruptures and imbrication of the lateral ligaments. Conversely, the purpose of non-anatomic reconstruction is to regain stability through various tenodesis techniques.

This study aims to examine the results of a treatment algorithm based on the integrity of the CFL and to investigate the significance of clinical examination, imaging modalities, arthroscopy, and anatomic reconstruction in highly active patients with chronic ankle instability.

## Materials and methods

We assessed 67 patients who were treated for chronic lateral ankle instability. All participants were recreational athletes or active service military personnel. Recurrent inversion injuries caused pain, swelling, stiffness, and a feeling of giving way, thus preventing participants from participating in sports as well as the physical aspects of military training exercises. The inclusion criteria for participating in this study were (1) pain, (2) instability, and (3) recurrent sprains that persisted after six months of intensive physiotherapy. Approval for this study was obtained from the Institutional Review Board of the hospital where the study was conducted.

The following exclusion criteria were used: (1) history of generalized ligamentous laxity, (2) history of neuromuscular disorders, (3) prior surgery of the contralateral ankle, (4) injuries at the contralateral ankle, (5) history of previous fractures of the involved ankle, (6) prior surgery other than anatomic reconstruction, tenodesis, or simple ankle arthroscopy by other surgeons, and (7) evidence of severe ankle arthritis.

Seven patients sought professional help for their instability elsewhere before our consultation. Among them, one patient was elected to address chronic ankle instability with Karlsson anatomic reconstruction, whereas six patients underwent ankle arthroscopy.

In preoperative assessment, a detailed history was taken and a thorough clinical examination was performed. Fractures, syndesmotic injuries, posttraumatic arthritis, loose bodies, bony avulsions, and osteophytes were identified using plain radiographs.

For the investigation of cartilage lesions, bone bruising, and peroneal tendon tears, ankle MRI without contrast was performed on all participants [12]. Furthermore, ankle MRI offered valuable additional information about the severity of ligamentous and cartilaginous injuries [7].

In the operating room, a specific surgeon performed provocative tests and standardized stress views. The integrity of the ATFL was assessed with the anterior drawer test. This test was performed with the ankle held plantar-flexed at 15 degrees. When talar translation was measured to be more than 4 mm or was at least 3 mm greater than the contralateral side, the test was considered positive.

The integrity of the CFL was evaluated clinically with the talar tilt test. This test was performed with the ankle held at 15 degrees of plantar-flexion, the talus in extreme varus, and the tibia rotated internally at 10 degrees. Stress views were taken and the tibiotalar angle was measured. The test was considered positive for angles greater than 10 degrees or when the tibiotalar angle difference was more than 6 degrees.

All patients underwent ankle arthroscopy. Its significance was twofold. It was used for recognizing those with CFL deficient ankles and for addressing associated intra-articular pathologies. It was, therefore, a valuable tool in determining whether ligament reconstruction was required, and if this proved not to be the case, it was used as a standalone treatment option [13,14]. We accepted arthroscopy as the gold standard for assessing the integrity of the ATFL and CFL [13].

We evaluated our outcomes using the American Orthopaedic Foot and Ankle Society (AOFAS clinical rating system) [15] and Tegner activity level score [16]. Both scores were calculated preoperatively and two years postoperatively.

Treatment algorithm

**Figure 1:**
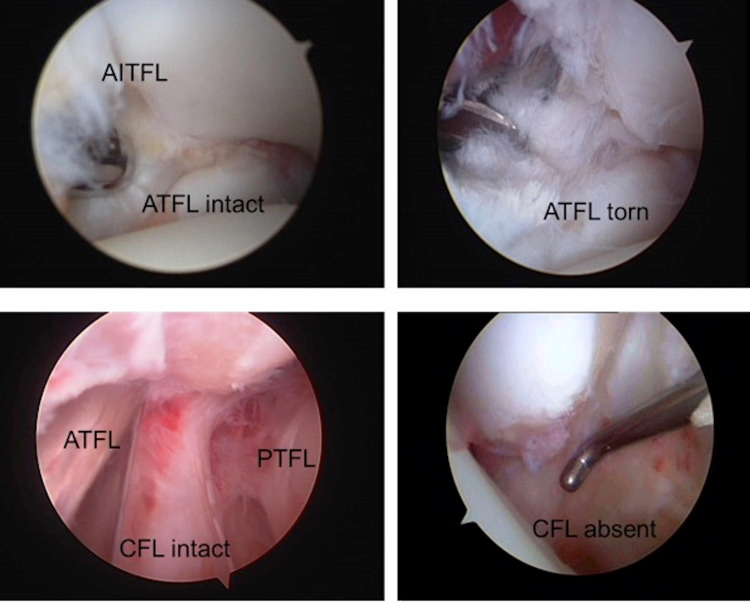
Arthroscopic view of the lateral aspect of the ankle. AITFL, anterior inferior tibiofibular ligament; ATFL, anterior talofibular ligament; PTFL, posterior talofibular ligament; CFL, calcaneofibular ligament

Ankle arthroscopy was performed in all patients. With the assistance of a GUHL Ankle Distractor (ACUFEX, Smith & Nephew Inc., Andover, MA, USA), two standard portals were made (anteromedial and anterolateral). Occasionally, a third anterocentral portal was also used. As part of our routine, the lateral ankle ligaments were evaluated. Concomitant intra-articular pathology was addressed accordingly. Cartilage defects were treated with debridement or microfracture technique. When needed, we also performed osteophyte resection, loose body excision, scar tissue removal, and synovectomy. In cases where the CFL was intact (group A) (Figure 1), no further treatment was performed. Patients were given a comprehensive physiotherapy program and were discharged on the day of surgery with instructions to ambulate with partial weight-bearing.

**Figure 2:**
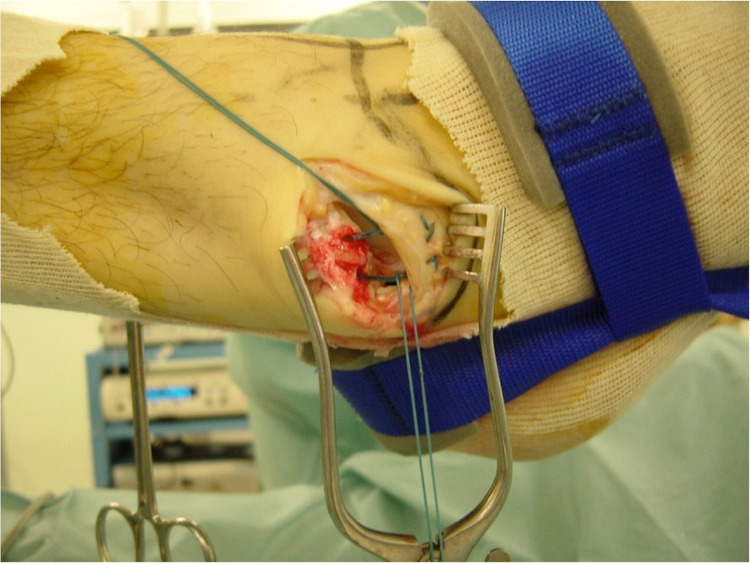
Insertion of two bone anchors to the fibula and reattachment of the lateral ligaments with non-absorbable sutures.

**Figure 3:**
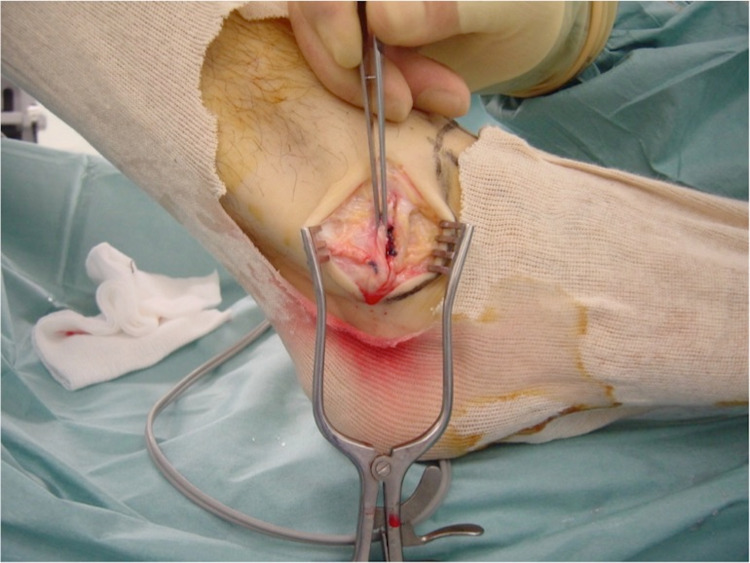
Inferior extensor retinaculum also tagged to the fibula.

In cases where the CFL was torn or avulsed (group B) (Figure 1), the modified Broström-Gould anatomic reconstruction was performed [17]. Ankle traction was removed and a 4-cm incision was made, starting from the anterior margin of the fibula and ending downwards and posteriorly, approximately 1 cm distal from the tip of the lateral malleolus. The lateral capsuloligamentous complex was divided. Care was taken not to damage the intermediate cutaneous branch of the superficial peroneal nerve anteriorly. The peroneal tendons and sural nerve were to be protected at the posterior end of the incision. The periosteum was stripped off from the tip of the lateral malleolus with the assistance of a bone rongeur. Two Mitek G-II anchors (Mitek Products Inc., Norwood, MA, USA) were then inserted into the bare bone area accommodating the reattachment of the CFL to the lateral malleolus. Moreover, the ATFL was incised, pulled distally, and attached with the CFL using the same anchor sutures. During the reattachment, the ankle was held reduced in a slightly everted position. Finally, the lateral portion of the inferior extensor retinaculum was tagged to the periosteum of the fibula with absorbable sutures (Figures 2, 3). Stability of the fixation was evaluated with the anterior drawer and talar tilt tests. Once it was ensured that ankle stability was reestablished, the wound was closed with absorbable sutures. Due to the CFL injuries occurring with the ankle in dorsiflexion, we elected to apply a below-knee cast with the ankle in a mildly plantar-flexed and valgus position for two weeks. DVT (deep vein thrombosis) prophylaxis was prescribed until the cast was removed. Toe-touch weight-bearing was allowed with the assistance of crutches. The patients had an overnight stay for pain management. They were then supplied with a detailed physiotherapy program and were discharged a day after surgery.

Rehabilitation

Group A (arthroscopy): Rehabilitation would not start before the second postoperative week. Between the second and fifth week, patients were advised to perform passive and active range of motion, progressive resistance exercises, and intrinsic muscle strengthening. Over the next two weeks, patients were allowed to perform proprioception, resistance, and closed chain training exercises. Finally, during weeks 9 to 12, patients were allowed to perform sports specific training.

Group B (capsule ligamentous reconstruction): During the first two postoperative weeks, a below-knee cast was applied. This cast was substituted with an Aircast boot during the third week when patients began leg cycling exercises. Between the third and sixth postoperative weeks, instructions were given for a transition from partial to full weight-bearing. During this period, isometric and progressively active exercises commenced. Also, patients were encouraged to start proprioception and manual resistance exercises, to focus on intrinsic muscle strengthening, and begin swimming.

Between weeks 6 and 12, closed chain as well as passive and active range of motion exercises were added to the rehabilitation program. Finally, between the third and sixth postoperative month, patients would gradually start sports specific exercises. The use of either strapping or bracing was recommended during rehabilitation, since both provide ankle support and hence decrease the risk of re-injury.

Evaluation

The follow-up period was two years. Patients were reviewed at 3, 6, 12, and 24 months after surgery at the outpatient clinic. During the 24-month review, we used the Clinical Rating System for the Ankle Hindfoot (AOFAS) and Tegner Activity Level Score for the clinical assessment and patient activity evaluation, respectively. The Ankle Hindfoot AOFAS rating system was preferred because the ankle and hindfoot were perceived as a single albeit complex system. Moreover, despite being originally designed for knee evaluation, Tegner Activity Level scoring system has also been used for the assessment of the ankle joint [15,16].

Data were expressed as mean ± SD for quantitative variables and as frequencies and percentages for qualitative variables. The Kolmogorov-Smirnov test was utilized for normality analysis of the quantitative variables.

Comparison of repeated measurements documented during preoperative assessment and 24-month postoperative review was performed using the paired samples Student’s t-test or Wilcoxon test (in case of violation of normality). The Mann-Whitney test was used for unpaired samples. The chi-square test was used to compare dichotomous variables. We used 95% confidence intervals. All tests were two-sided, with statistical significance set at p < 0.05.

Cohen's kappa coefficient was used to determine the inter-rater reliability between clinical examination and arthroscopy, as well as MRI and arthroscopy. Strength of the kappa coefficients was interpreted in the following manner: 0.01 to 0.20: slight; 0.21 to 0.40: fair; 0.41 to 0.60: moderate; 0.61 to 0.80: substantial; 0.81 to 1.00: near perfect.

All analyses were carried out using SPSS Version 21 (IBM Corp., Armonk, NY, USA).

## Results

Out of 67 patients, 58 were males and 9 were females. The mean age was 29.9 ± 6.4 years (range: 13-50 years). The right side was affected in 39 (39/67; 58.2%) cases and the left side in 28 (23/67; 41.8%) cases. Sports injuries were the cause for ankle instability in 44 (44/67; 65.7%) cases, whereas trauma suffered during military training was the cause in 23 (23/67; 34.3%) cases.

The mean number of recurrent inversion injuries per patient was 1.0 ± 2.1 (range: 1-10), whereas the median duration of symptoms (interval between first injury and surgery) was 23 months (range: 6-168 months).

**Figure 4:**
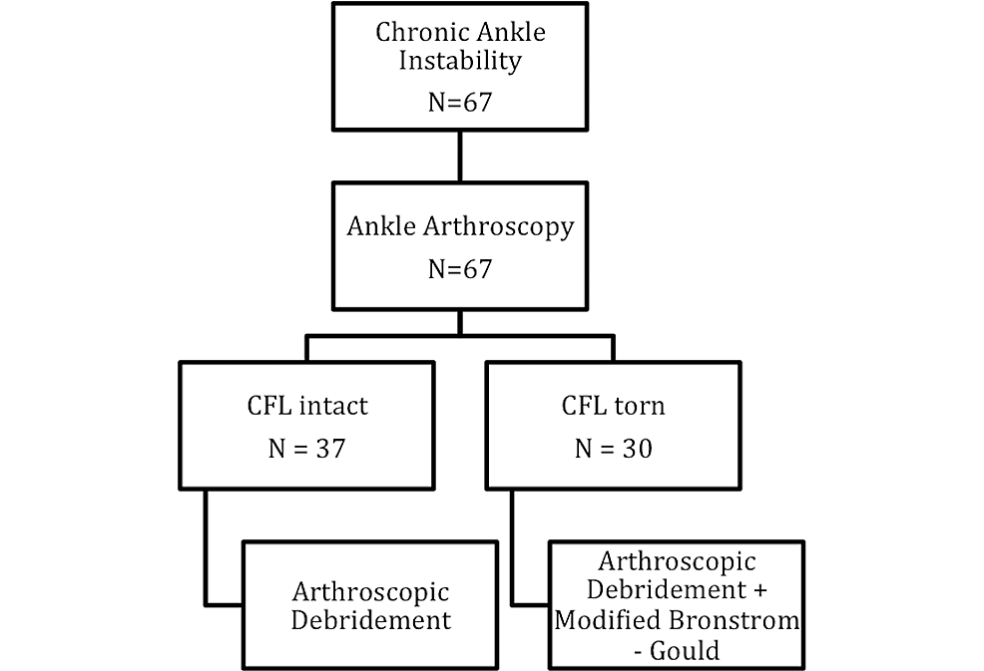
All patients underwent ankle arthroscopy, of whom 30 patients were diagnosed with CFL tears. They were addressed further with the modified Broström–Gould procedure. CFL, calcaneofibular ligament

We restricted ankle arthroscopy to 37 patients (37/67 [55.22%], group A). The remaining 30 patients (30/67 [44.78%], group B) developed mechanical instability with combined ATFL and CFL rupture. They underwent ankle arthroscopy followed by modified Broström-Gould reconstruction (Figure 4).

During arthroscopy, osteophytes were encountered and resected in 14 patients in Group A and eight patients in group B. Loose bodies were removed from 11 patients in group A and eight in group B. Cartilage lesions were documented and debrided in 13 patients in group A and 10 patients in group B, whereas synovitis and scar tissue were evident in 30 (30/67; 81.08%) group A patients. In Group B, 15 patients had the former findings. Synovitis and scar tissue formation were more common in group A (p = 0.011). Mild syndesmotic attenuation was evident in three patients in group A and four patients in group B, whereas deltoid ligament injury was prevalent in three patients in group A and two patients in group B. No PTFL ruptures were identified in either group. No postoperative ankle instability or relapsing ankle sprain was reported in either group.

Despite their previous high level of activity, our studied population demonstrated a very low post-injury mean Tegner score of 3.62 ± 1.29 (range: 0-10). The latter improved to 6.2 ± 2.15 two years after surgery (p = 0.009). In particular, the mean post-injury Tegner score was 3.48 ± 1.49 in group A and 3.61 ± 1.29 in group B, whereas at two years post-surgery it increased to 6.12 ± 2.03 (p = 0.008) and 6.2 ± 2.15 (p = 0.009), respectively. The preoperative AOFAS mean score was 68.9 ± 14.19 for both groups. Two years after surgery, it improved to 96.6 ± 6.84 in group A and 96.9 ± 6.53 in group B, whereas the overall mean score was 96.9 ± 6.54 (p < 0.001) (Table 1).

**Table 1:** Tegner and AOFAS scores increased significantly in both groups at 24 months after surgery (paired samples t-test). AOFAS, American Orthopaedic Foot and Ankle Society

	Group A (n=37)		Group B (n=30)	
AOFAS score	Mean	SD	Mean	SD
Preoperative	68.9	14.19	68.9	14.19
24 months post-operatively	96.6	6.84	96.9	6.53
p-Value	p < 0.001		p < 0.001	
Tegner score	Mean	SD	Mean	SD
Preoperative	3.48	1.49	3.61	1.29
24 months post-operatively	6.12	2.03	6.2	2.15
p-Value	p < 0.008		p < 0.009	

The sensitivity and specificity of MRI in detecting CFL ruptures were 46.7% and 87%, respectively. In contrast, clinical examination was 47.4% sensitive and 100% specific. Moreover, when performed under anesthesia, clinical examination exhibited 93.3% sensitivity and 100% specificity. Inter-rater reliability for CFL tears was very high for clinical examination under anesthesia in conjunction with arthroscopy (k = 0.909), moderate between clinical examination and ankle arthroscopy (k = 0.514), and fair between MRI and ankle arthroscopy (k = 0.357).

No major postoperative complications were documented regardless of the type of treatment. Six patients from each group developed postoperative synovitis that necessitated treatment with secondary arthroscopic synovectomy. One patient from group A developed superficial peroneal nerve intermediate branch entrapment. The former was attributed to scar tissue formation around the anterolateral portal and was treated successfully with local corticosteroid injections. Three patients from group B complained of skin irritation. This was a consequence of bulky suture knots during lateral capsuloligamentous reconstruction. The complication was addressed with surgical excision of the knots.

No postoperative residual swelling was identified. Postoperative stiffness developed in two patients in group A, with severe restriction of ankle dorsiflexion (greater than 15 degrees). However, both patients saw great improvement after intensive physiotherapy.

## Discussion

Our treatment algorithm for chronic ankle instability highlighted the role of arthroscopy and lateral capsuloligamentous complex repair in high-demand groups.

Ankle arthroscopy confirmed the presence of cartilaginous lesions, osteophytes, and loose bodies, as outlined previously by other imaging modalities and clinical examination.

It also helped identify synovitis and scar tissue that could not be viewed by MRI scans [6]. These findings had a higher prevalence in group A (p = 0.011) and most likely stemmed from post-injury hematomas. Moreover, relevant research suggested that their presence was associated with incomplete or defective ATFL healing [18] and anterolateral ankle impingement syndrome [19].

Conversely, the greater severity of soft tissue damage and the substantial separation between the stumps of the torn ligaments in group B may be related to the lower incidence of synovitis and scar tissue. Thus, the absence of scar tissue could be indicative of a lower healing potential in patients with CFL tears and may justify the need for further ligament reconstruction.

Ankle arthroscopy provided useful information about the pattern of the injury and decision-making regarding the necessity for LLC reconstruction. It also permitted the management of associated intra-articular injuries that were responsible for the majority of symptoms seen in patients with functional chronic ankle instability [13]. The postoperative results in group A indicated that arthroscopic treatment alone sufficed for the management of patients with functional instability. Hence, to fully support this observation, a double-blinded clinical study must be designed.

In patients with CFL tear, our algorithm mandated further treatment. Over the years, there have been several anatomic and non-anatomic techniques describing the reconstruction or repair of the lateral ankle ligaments.

Among the disadvantages of non-anatomic reconstruction techniques are altered kinematics, decreased subtalar and talocrural joint mobility, increased risk for adjacent cutaneous nerve injury, and the sacrifice of the peroneus brevis [20-22]. All of the above are associated with poor postoperative outcome and higher risk for ankle arthritis.

In contrast, the rationale for anatomic repair is to restore normal anatomy by imbrication of the lateral capsuloligamentous complex. The reconstruction technique described by Broström [11] is considered the cornerstone of anatomic repair. It has a simple surgical approach and does not affect the talocrural and subtalar motion. Its most serious complication is superficial peroneal or sural nerve injury. However, these are markedly rare complications that are more common during non-anatomic reconstruction.

Broström reconstruction is associated with lower morbidity, quicker functional recovery, and no proprioception interference [23]. Nevertheless, its value in chronic ligament tears is debatable. Karlsson et al. [24] reported poor results in patients with generalized ligamentous laxity, long-standing ligamentous insufficiency, or previous operation.

There have been many efforts to address the limitations of Broström technique. A modification described by Gould [17] proposed that additional mobilization and reattachment of the lateral portion of the extensor retinaculum to the fibula would offer greater talocrural and subtalar joint stability. Indeed, this modification resulted in reduced anterior talar displacement and a decreased talar internal rotation [25].

Most studies that evaluated the outcomes of anatomic reconstruction or tenodesis examined diverse groups in terms of activity and athletic level. However, recent data demonstrated that modified Broström-Gould reconstruction offered satisfactory results even in groups of professional ballet dancers, recreational artists, high-demand athletes, and military personnel [26-28].

Assessing such groups is rather interesting since the functional demands of the latter are substantially higher compared to people living a sedentary lifestyle. These demands lead to a proportionately higher threshold for expectations. Thus, while studying excessively active individuals, we frequently face the paradox of improved functional postoperative scores coexisting with low postoperative patient satisfaction.

Some authors highlighted the role of the ATFL in rotational ankle microinstability and the need for its repair when a tear is present [18,19]. Conversely, we postulated that the CFL was the primary restraint for lateral ankle instability and that reconstruction of the lateral capsuloligamentous complex should be offered only to CFL-deficient patients.

The scores used for evaluating the postoperative outcome and level of activity indicated that both groups improved significantly. AOFAS scores measured during the 24-month follow-up demonstrated that most patients reported minor to no limitations regarding their fitness status (p < 0.001). Furthermore, despite the markedly low post-injury Tegner score, all participants improved considerably and ultimately became fit to return to heavy labor and competitive sports (p = 0.009). Therefore, the excellent postoperative outcome documented in both groups supports our hypothesis regarding the lower clinical significance of ATFL.

We also demonstrated that MRI scans and clinical examination lacked sensitivity for diagnosing LLC injuries, even though clinical examination was highly specific. These findings agree with other studies citing that MRI has limitations in diagnosing CFL injuries [29,30]. Clinical examination under anesthesia proved to be highly sensitive and specific. Its high inter-rater reliability in conjunction with ankle arthroscopy corroborates that it is a highly efficient diagnostic tool for the assessment of chronic ankle instability. Regardless, examination under anesthesia remains impractical since it can only be performed in the operating room before arthroscopy.

To our knowledge, this study has the highest number of extremely active patients assessed for chronic lateral ankle instability. However, as this is a retrospective study, the need for a superior level of evidence calls for the design of a prospective study with a higher number of participants and increased (more than 80%) statistical power.

## Conclusions

Our proposed algorithm offers a reliable method for accurate evaluation and successful treatment of chronic lateral ankle instability. This is essential for high-demand patients like athletes and combat-ready military personnel. We therefore recommend a treatment algorithm for chronic ankle instability depending on arthroscopic findings regarding the integrity of CFL.
